# Hyperopic reserve insufficiency and myopic pre-disposition in pre-school children aged 3–6 years: a large-scale cross-sectional survey

**DOI:** 10.3389/fmed.2026.1915177

**Published:** 2026-08-10

**Authors:** Dan Jia, Yali Chen, Yunteng Wang, Huiying Lan, Yuxiang Zhou, Min Ouyang

**Affiliations:** Department of Ophthalmology, Ganzhou Maternal and Child Health Hospital, Ganzhou, Jiangxi, China

**Keywords:** associated factors, axial length, hyperopic reserve insufficiency, myopia susceptibility, pre-myopia, pre-school children

## Abstract

**Objective:**

To estimate the prevalence of hyperopic reserve insufficiency (HRI) and examine its associated factors among pre-school children aged 3–6 years in Ganzhou, China.

**Methods:**

This cross-sectional study included 3,360 pre-school children aged 3–6 years. All participants underwent cycloplegic autorefraction and axial length (AL) measurement. HRI was defined using age-specific spherical equivalent refraction (SER) thresholds. SER was also used to describe age-specific refractive status and to conduct a sensitivity analysis using the International Myopia Institute (IMI) pre-myopia criterion (−0.50 D < SER ≤ +0.75 D). Guardian-completed Questionnaires assessed paternal/maternal high myopia, outdoor time, near-work, and residence. Multivariable logistic regression was performed with HRI coded as 1 and sufficient hyperopic reserve coded as 0.

**Results:**

HRI was identified in 380 children (11.3%, 95% CI: 10.3–12.4%). Age-specific HRI prevalence increased from 4.1% (95% CI: 2.8–6.0%) at age 3 to 27.5% (95% CI: 23.9–31.5%) at age six. Mean SER declined from +2.35 ± 0.37 D at age 3 to +1.05 ± 0.54 D at age 6. IMI-defined pre-myopia was found in 179 (5.3%). In multivariable analysis, older age (OR = 1.86), longer AL (OR = 1.70), paternal (OR = 1.28) and maternal (OR = 1.31) high myopia, near-work (OR = 1.06), and urban residence (OR = 1.40) were associated with higher HRI odds. Male sex was protective (OR = 0.75, 95% CI: 0.60–0.94). Outdoor time showed lower odds but was non-significant (OR = 0.93, 95% CI: 0.85–1.01). IMI sensitivity analysis showed consistent directions.

**Conclusions:**

HRI was detectable in a notable proportion of pre-schoolers and was accompanied by an age-related decline in SER. HRI was associated with age, AL, parental myopia, residence, and early-life visual behaviors. Due to the cross-sectional design, findings are associational, not causal.

## Introduction

Childhood myopia is a major global public health challenge, and its prevalence is particularly high and still increasing in East Asia (1, 2). Earlier onset of myopia is associated with a longer duration of progression and a greater lifetime risk of high myopia and sight-threatening complications, including retinal detachment, myopic maculopathy, and glaucoma (3–5). Therefore, identifying children who may already show unfavorable refractive trajectories before manifest myopia is clinically and public-health relevant.

Hyperopic reserve refers to the physiological hyperopic refractive status present during early childhood and is regarded as a buffer against early myopia onset (6–9). During normal emmetropization, hyperopic reserve gradually decreases as ocular growth proceeds. Children with insufficient reserve may be closer to the pre-myopic range, a refractive state proposed by the International Myopia Institute (IMI) to indicate an increased likelihood of future myopia when accompanied by other risk factors (7, 10, 11).

The depletion of hyperopic reserve is likely influenced by both biological and environmental factors. Axial elongation is a structural driver of refractive change, whereas familial susceptibility, outdoor exposure, near-work behavior, and urban living environments may also contribute to early refractive development (12–17). However, large pre-school studies simultaneously evaluating biometric, familial, behavioral, and residential factors remain limited.

The present study aimed to estimate the prevalence of HRI among children aged 3–6 years in Ganzhou, China, and to examine its associations with AL, parental high myopia, outdoor time, study-related near-work time, sex, and residence. Given the cross-sectional design, the study was intended to identify associated factors rather than determine causal effects or predict future myopia onset.

## Methods

### Study design and participants

This cross-sectional epidemiological study was conducted among pre-school children aged 3–6 years in Ganzhou, Jiangxi Province, China. Children were recruited from kindergartens using stratified cluster sampling to improve representation across age, sex, and urban-rural residence. The final de-identified analytic dataset did not retain kindergarten, class, or sampling-weight identifiers; therefore, design-weighted or cluster-specific models could not be implemented. This limitation is acknowledged below.

Participants were included if they were aged 3.0–6.9 years, had written informed consent from a parent or legal guardian, and could cooperate with cycloplegic refraction and AL measurement. Children with organic ocular disease, previous ocular trauma or surgery, strabismus, amblyopia, or neurological or developmental conditions that could affect ophthalmic examination were excluded. Children with fewer than three reliable measurements for autorefraction or AL were also excluded.

### Sample size and precision

No formal *a priori* sample-size calculation was performed specifically for the present HRI analysis. The final sample size was determined by the available eligible children who completed the pre-school vision screening and met the study criteria. With 3,360 participants and an observed HRI prevalence of 11.3%, the estimated 95% CI for the overall prevalence was 10.3 to 12.4%, providing an absolute precision of approximately ±1.1 percentage points. This *post hoc* precision estimate is descriptive and should not be interpreted as an *a priori* power calculation.

### Ophthalmic examination

All participants underwent standardized ophthalmic examination by trained ophthalmologists. Cycloplegia was induced using three drops of 0.5% tropicamide administered at 5-min intervals. Pupillary light reflex was assessed 30 min after the first drop; if the cycloplegic response was considered incomplete, an additional drop was administered before autorefraction. Cycloplegic autorefraction was performed using a table-mounted autorefractor (Topcon KR-8900, Topcon Corporation, Tokyo, Japan). AL was measured with a non-contact optical biometer (SOVI Biometer, SOVI, China). For each eye, three consecutive measurements were obtained and averaged.

Both eyes underwent cycloplegia and examination as part of the clinical screening protocol, allowing clinicians to identify binocular abnormalities and confirm eligibility. To avoid within-child correlation in the statistical analysis, right-eye data were used preferentially for the primary analysis, and left-eye measurements were substituted only when right-eye measurements did not meet quality-control requirements. The de-identified analytic dataset did not retain a separate flag for left-eye substitution, which is acknowledged as a limitation.

### Definition of hyperopic reserve insufficiency

HRI was defined according to age-specific SER thresholds based on national pediatric ophthalmology recommendations: SER ≤ +2.00 D at age 3 years, ≤ +1.50 D at ages 4–5 years, and ≤ +1.00 D at age 6 years. SER was calculated as spherical power plus one-half cylindrical power during ophthalmic data processing. Children not meeting these criteria were classified as having sufficient hyperopic reserve. For descriptive refractive classification, IMI-defined pre-myopia was defined as −0.50 D < SER ≤ +0.75 D, myopia as SER ≤ −0.50 D, and likely persistent hyperopia as SER ≥ +3.00 D. A sensitivity analysis was conducted by modeling IMI-defined pre-myopia as the outcome among non-myopic children.

### Parental high myopia and lifestyle factors

Information on paternal and maternal high myopia, outdoor time, study-related near-work time, and residence was obtained using guardian-completed questionnaires. Paternal and maternal high myopia were defined as self-reported high myopia in the corresponding parent, coded as 0 = no and 1 = yes. Outdoor time was defined as the average daily time spent outdoors. Study-related near-work time was defined as the average daily time spent in near-work activities related to reading, writing, drawing, screen-based learning, or other pre-school study activities. Residence was classified as urban or rural according to the child's registered or long-term residential location reported by the guardian.

### Statistical analysis

Continuous variables with approximately symmetric distributions, including SER and AL, are presented as mean ± standard deviation (SD), whereas outdoor time and study-related near-work time are presented as median and interquartile range (IQR) because of skewed distributions. Categorical variables are presented as number and percentage. Comparisons between children with HRI and those with sufficient reserve were performed using Welch's *t*-test for normally distributed continuous variables, the Mann–Whitney *U*-test for skewed continuous variables, and the chi-square test for categorical variables. Comparisons across age groups were performed using one-way ANOVA for SER and AL, Kruskal–Wallis tests for skewed lifestyle variables, and chi-square tests for categorical variables.

The multivariable logistic regression outcome was recoded as HRI = 1 and sufficient hyperopic reserve = 0, so that ORs greater than 1 indicate higher odds of HRI. Covariates included age, sex, paternal high myopia, maternal high myopia, AL, outdoor time, study-related near-work time, and residence. A sensitivity logistic regression model used IMI-defined pre-myopia as the outcome among non-myopic children. Because sampling weights and cluster identifiers were unavailable in the de-identified analytic dataset, models used heteroskedasticity-robust (HC3) standard errors as a conservative approach to reduce sensitivity to model-based standard-error assumptions. Multicollinearity was evaluated using variance inflation factors (VIFs), with VIF < 5 considered acceptable. Model discrimination was assessed using the area under the receiver operating characteristic curve (AUC), and calibration was assessed using the Hosmer–Lemeshow test. A two-sided *P*-value < 0.05 was considered statistically significant. All analyses were conducted using SPSS version 26.0 and R version 4.2.2.

### Ethics statement

This study adhered to the principles of the Declaration of Helsinki and was approved by the Ethics Committee of Ganzhou Maternal and Child Health Hospital (Approval No. 2024-LS-136). Written informed consent was obtained from the parent or legal guardian of each participant before enrollment.

## Results

### Demographic characteristics and HRI prevalence

A total of 3,360 pre-school children aged 3–6 years were included. The mean age was 4.44 ± 0.97 years, and 1,972 participants (58.69%) were boys. HRI was identified in 380 children, corresponding to an overall prevalence of 11.3% (95% CI: 10.3–12.4%). The mean SER was +1.74 ± 0.57 D, and the mean AL was 22.99 ± 1.09 mm. IMI-defined pre-myopia was present in 179 children (5.33%), myopia in eight children (0.24%), and SER ≥ +3.00 D in 40 children (1.19%). Paternal and maternal high myopia were reported in 31.61% and 33.81% of children, respectively. Median outdoor time and study-related near-work time were 0.60 (IQR: 0.50–1.70) h/day and 0.60 (IQR: 0.50–2.50) h/day, respectively. Urban residents accounted for 52.02% of the sample (Table 1).

**Table 1:** Baseline characteristics of study participants.

Variable	Value
HRI, *n* (%)	380 (11.31)
Sufficient hyperopic reserve, *n* (%)	2,980 (88.69)
Overall HRI prevalence, % (95% CI)	11.3 (10.3–12.4)
SER (D), mean ± SD	1.74 ± 0.57
IMI-defined pre-myopia, *n* (%)	179 (5.33)
Myopia, *n* (%)	8 (0.24)
SER ≥+3.00 D, *n* (%)	40 (1.19)
Age (years), mean ± SD	4.44 ± 0.97
Male sex, *n* (%)	1,972 (58.69)
Height (cm), mean ± SD	112.04 ± 16.00
Weight (kg), mean ± SD	18.84 ± 4.56
Paternal high myopia, *n* (%)	1,062 (31.61)
Maternal high myopia, *n* (%)	1,136 (33.81)
Axial length (mm), mean ± SD	22.99 ± 1.09
Outdoor time (h/day), median (IQR)	0.60 (0.50, 1.70)
Study-related near-work time (h/day), median (IQR)	0.60 (0.50, 2.50)
Urban residence, *n* (%)	1,748 (52.02)

Data are presented as mean ± SD, median (IQR), or *n* (%), as appropriate. CI, confidence interval; HRI, hyperopic reserve insufficiency; IMI, international myopia institute; IQR, interquartile range; SER, spherical equivalent refraction.

### Age-specific distribution of HRI and related characteristics

Age-specific HRI prevalence increased from 4.1% (95% CI: 2.8–6.0%) at age 3 years to 27.5% (95% CI: 23.9–31.5%) at age 6 years (*P* < 0.001). Mean SER decreased progressively from +2.35 ± 0.37 D at age 3 years to +1.05 ± 0.54 D at age 6 years (*P* < 0.001), while AL increased from 22.62 ± 1.03 mm to 23.43 ± 0.99 mm (*P* < 0.001). IMI-defined pre-myopia also became more common with age, increasing from 0.3% at age 3 years to 21.0% at age 6 years. Outdoor time and study-related near-work time differed across age groups (Table 2).

**Table 2:** Age-specific HRI prevalence and related characteristics.

Age group	*n*	SER (D), mean ±SD	HRI, *n* (%; 95% CI)	IMI pre-myopia, *n* (%)	Myopia, *n* (%)	SER ≥+3.00 D, *n* (%)	AL (mm), mean ±SD	Outdoor time, median (IQR)	Study time, median (IQR)
3 years	630	2.35 ± 0.37	26 (4.1; 95% CI 2.8–6.0)	2 (0.3)	0 (0.0)	34 (5.4)	22.62 ± 1.03	0.60 (0.50, 1.90)	0.55 (0.50, 1.80)
4 years	1,166	1.78 ± 0.39	86 (7.4; 95% CI 6.0–9.0)	23 (2.0)	0 (0.0)	4 (0.3)	22.88 ± 1.08	0.60 (0.50, 1.90)	0.60 (0.50, 2.20)
5 years	1,030	1.67 ± 0.43	121 (11.7; 95% CI 9.9–13.9)	42 (4.1)	1 (0.1)	2 (0.2)	23.13 ± 1.07	0.60 (0.50, 1.70)	0.60 (0.50, 2.60)
6 years	534	1.05 ± 0.54	147 (27.5; 95% CI 23.9–31.5)	112 (21.0)	7 (1.3)	0 (0.0)	23.43 ± 0.99	0.60 (0.50, 1.10)	0.70 (0.50, 4.17)
*P-value*	—	< 0.001	< 0.001	< 0.001	< 0.001	< 0.001	< 0.001	0.047	< 0.001

*P*-values were obtained using chi-square tests for categorical variables, one-way ANOVA for SER and AL, and Kruskal–Wallis tests for outdoor and study-related near-work time. IMI pre-myopia was defined as −0.50 D < SER ≤ +0.75 D.

### Comparison between children with and without HRI

Compared with children with sufficient hyperopic reserve, children with HRI were older, had lower SER, and had longer AL. They also had higher proportions of paternal high myopia, maternal high myopia, and urban residence. Outdoor time was lower in the HRI group. Study-related near-work time had a wider upper IQR in the HRI group, although the between-group comparison did not reach statistical significance using the Mann–Whitney *U*-test (Table 3).

**Table 3:** Comparison of characteristics between children with HRI and those with sufficient hyperopic reserve.

Characteristic	HRI (*n* = 380)	Sufficient reserve (*n* = 2,980)	*P-value*
Age (years), mean ± SD	5.02 ± 0.94	4.36 ± 0.95	< 0.001
SER (D), mean ± SD	0.72 ± 0.53	1.87 ± 0.43	< 0.001
Height (cm), mean ± SD	111.15 ± 13.35	112.15 ± 16.31	0.178
Weight (kg), mean ± SD	18.97 ± 5.05	18.82 ± 4.49	0.597
Axial length (mm), mean ± SD	23.60 ± 0.95	22.92 ± 1.08	< 0.001
Outdoor time (h/day), median (IQR)	0.50 (0.50, 0.70)	0.60 (0.50, 1.90)	< 0.001
Study-related near-work time (h/day), median (IQR)	0.60 (0.50, 5.40)	0.60 (0.50, 2.40)	0.398
Male sex, *n* (%)	202 (53.2)	1,770 (59.4)	0.023
Paternal high myopia, *n* (%)	139 (36.6)	923 (31.0)	0.031
Maternal high myopia, *n* (%)	148 (38.9)	988 (33.2)	0.028
Urban residence, *n* (%)	219 (57.6)	1,529 (51.3)	0.023

P-values were obtained using Welch's t-test, Mann–Whitney U-test, or chi-square test, as appropriate.

### Multivariable analysis

The coding scheme for categorical variables is shown in Table 4. In the primary multivariable model, HRI was coded as the dependent variable (HRI = 1; sufficient hyperopic reserve = 0). Older age, longer AL, paternal high myopia, maternal high myopia, longer study-related near-work time, and urban residence were associated with higher odds of HRI. Male sex was associated with lower odds of HRI. Outdoor time showed a lower estimated odds of HRI but did not reach conventional statistical significance after HC3 robust standard-error adjustment. All VIF values were below 1.1, indicating no evidence of problematic multicollinearity (Table 5). The model showed acceptable discrimination (AUC = 0.751) and no evidence of poor calibration by the Hosmer–Lemeshow test (*P* = 0.255). In the IMI sensitivity model among non-myopic children (*n* = 3,352), IMI-defined pre-myopia was identified in 179 children (5.3%). Older age, longer AL, study-related near-work time, and urban residence were associated with higher odds of IMI-defined pre-myopia, while other variables showed broadly similar directions but did not all reach statistical significance (Table 5).

**Table 4:** Variable coding for logistic regression analysis.

Variable	Coding
Hyperopic reserve insufficiency	0 = sufficient hyperopic reserve; 1 = HRI
Sex	0 = female; 1 = male
Paternal high myopia	0 = no; 1 = yes
Maternal high myopia	0 = no; 1 = yes
Residence	0 = rural; 1 = urban

The dependent variable was recoded as HRI = 1 and sufficient hyperopic reserve = 0.

**Table 5:** Sensitivity logistic regression analysis using IMI-defined premyopia as the outcome.

Independent variable	Estimate	Robust SE	z value	OR (95% CI)	*P*-value
Age (years)	1.319	0.129	10.207	3.74 (2.90–4.82)	< 0.001
Sex (male)	−0.290	0.171	−1.696	0.75 (0.53–1.05)	0.090
Paternal high myopia	0.150	0.180	0.834	1.16 (0.82–1.65)	0.405
Maternal high myopia	0.322	0.178	1.807	1.38 (0.97–1.96)	0.071
Axial length (mm)	1.091	0.102	10.718	2.98 (2.44–3.63)	< 0.001
Outdoor time (h/day)	−0.099	0.065	−1.523	0.91 (0.80–1.03)	0.128
Study time (h/day)	0.135	0.035	3.894	1.14 (1.07–1.23)	< 0.001
Urban residence	0.403	0.174	2.313	1.50 (1.06–2.10)	0.021

The sensitivity model was fitted among non-myopic children (*n* = 3,352) with IMI-defined premyopia (−0.50 D < SER < = +0.75 D) coded as 1. Robust SEs were calculated using the HC3 method. Model AUC = 0.893; Hosmer-Lemeshow *P* = 0.382. IMI, International Myopia Institute; OR, odds ratio; SE, standard error; SER, spherical equivalent refraction.

## Discussion

This large cross-sectional study examined HRI in 3,360 pre-school children aged 3–6 years in Ganzhou, China. HRI was present in 11.3% of pre-school children and became more common with increasing age. SER declined across age groups, while AL increased progressively. Longer AL, paternal and maternal high myopia, urban residence, and study-related near-work time were associated with higher odds of HRI, whereas male sex was associated with lower odds. Outdoor time showed a lower estimated odds of HRI, but this association was not statistically significant in the model with robust standard errors. The IMI sensitivity analysis provided additional support for the association of age, AL, study-related near-work time, and residence with a more myopia-prone refractive profile. These findings should be interpreted as cross-sectional associations rather than causal or longitudinal effects.

### Age-related decline in hyperopic reserve

A marked age-related increase in HRI was observed across the 3–6-year age range, accompanied by a progressive decline in mean SER. This pattern is consistent with the process of emmetropization, in which physiological hyperopia decreases as the eye grows (6, 9, 18, 19). However, the interpretation of age requires caution because the HRI definition itself used age-specific SER thresholds. Therefore, age should not be interpreted as an independent biological cause of HRI; rather, it indicates that older pre-school children were more likely to fall below age-specific reserve thresholds and to approach the IMI-defined pre-myopia range.

### Axial length as an early structural marker

AL showed one of the strongest associations with HRI. Children with HRI had longer AL than those with sufficient reserve, and the association remained evident in multivariable and exploratory age-stratified analyses. This finding supports the view that axial elongation is a key structural correlate of early refractive change. Because AL can be measured objectively and non-invasively, combining AL with age-specific refractive screening may improve early identification of children with unfavorable refractive profiles (20, 21).

### Familial pre-disposition and parental high myopia

Both paternal and maternal high myopia were associated with higher odds of HRI. This is consistent with previous evidence that parental myopia and high myopia are related to childhood refractive development and hyperopic reserve (15, 22, 23). Nevertheless, parental high myopia was self-reported by guardians, and direct parental refraction was not performed. Misclassification is therefore possible and should be considered when interpreting the magnitude of these associations.

### Behavioral and environmental factors

Study-related near-work time and urban residence were associated with higher odds of HRI in the primary model and with IMI-defined pre-myopia in the sensitivity model. These associations should be interpreted as cross-sectional relationships rather than causal effects. Outdoor time had a lower estimated odds ratio but was not statistically significant in the robust primary model. This finding should not be interpreted as evidence against the potential relevance of outdoor exposure; instead, it may reflect measurement error, skewed exposure distribution, or residual confounding in questionnaire-derived lifestyle data (12, 13, 16, 24).

### Clinical and public-health implications

The findings suggest that HRI assessment may have value when interpreted together with AL, family history, and visual behavior information. Children whose refractive status is close to age-specific reserve thresholds may warrant closer follow-up, especially when they have longer AL or parental high myopia. However, because the present study is cross-sectional, the results should not be used to infer causality or to predict individual future myopia onset without longitudinal validation.

### Strengths and limitations

This study included a large pre-school sample and used cycloplegic SER-derived HRI classification together with AL measurement and questionnaire-derived familial and lifestyle variables. The revised statistical analysis also clarified the outcome direction, provided age-specific SER and refractive-category summaries, reported prevalence CIs, and added model diagnostics and an IMI sensitivity analysis.

Several limitations should be acknowledged. First, the cross-sectional design precludes causal inference and does not allow direct prediction of future myopia onset. Second, the dataset did not retain kindergarten, class, sampling-weight, or left-eye substitution indicators, preventing survey-weighted, cluster-specific, GEE, mixed-effects, or eye-substitution analyses. Third, paternal and maternal high myopia and lifestyle exposures were based on guardian report and may be subject to recall, reporting, and exposure misclassification. Fourth, the use of 0.5% tropicamide may provide less complete cycloplegia than stronger agents in some young children, potentially leading to underestimation of hyperopia and overestimation of HRI prevalence. Finally, the study was conducted in a single city, which may limit generalizability to populations with different socioeconomic, educational, or environmental contexts.

### Future directions

Future studies should prospectively retain binocular SER values, eye-specific measurement quality indicators, sampling-cluster information, and direct parental refraction where feasible. Longitudinal follow-up from pre-school to school age is needed to determine whether HRI, alone or combined with AL, SER trajectory, and family history, predicts subsequent myopia onset.

## Conclusion

HRI was present in 11.3% of pre-school children in Ganzhou and became more common with increasing age, alongside a progressive age-related decline in SER. In the multivariable analysis, older age, longer AL, paternal and maternal high myopia, study-related near-work time, and urban residence were associated with higher odds of HRI, whereas male sex was associated with lower odds. The IMI sensitivity analysis showed broadly consistent findings for age, AL, study-related near-work time, and residence. These findings support the potential value of combining refractive reserve status with biometric, familial, and behavioral information in pre-school vision screening. Given the cross-sectional design, the findings should be interpreted as associations that require longitudinal validation.

## Data Availability

The original contributions presented in the study are included in the article/supplementary material, further inquiries can be directed to the corresponding author.
